# Joint association of frailty and depression with new-onset digestive disease among elderly Chinese population

**DOI:** 10.3389/fnut.2025.1590194

**Published:** 2025-07-28

**Authors:** Fan Zhang, Yu-Jun Xiong, Xiang-Da Meng, Tian Lv, Du-Juan Yang

**Affiliations:** ^1^Department of Gastroenterology, Beijing Hospital, National Center of Gerontology, Institute of Geriatric Medicine, Chinese Academy of Medical Sciences, Beijing, China; ^2^Department of Hernia and Abdominal Wall Surgery, Peking University Peoples' Hospital, Beijing, China; ^3^Department of Neurology, Zhuji Affiliated Hospital of Wenzhou Medical University, Zhuji, China; ^4^Department of Geriatrics, Zhuji Affiliated Hospital of Wenzhou Medical University, Zhuji, China

**Keywords:** CHARLS, depression, digestive disease, frailty, mediation analysis

## Abstract

**Background:**

Digestive diseases impose a substantial global health burden, yet the joint impact of frailty and depression on their incidence remains underexplored.

**Methods:**

This cohort study analyzed 5,506 adults aged ≥ 65 years from the China Health and Retirement Longitudinal Study (2011–2018). Participants with baseline digestive diseases or missing data were excluded. Cox proportional hazards models assessed associations, while mediation analysis evaluated bidirectional roles of the frailty index (FI) and 10-item Center for Epidemiologic Studies Depression Scale (CESD-10) in new-onset digestive diseases.

**Results:**

Over 7 years, 988 participants developed digestive diseases. Frailty (HR = 1.66, *p* < 0.001) and depression (HR = 1.62, *p* < 0.001) independently increased risk, with the highest hazard in comorbid cases (HR = 2.16, *p* < 0.001). Frailty mediated 30.5% of depression’s effect, while depression mediated 45.2% of frailty’s impact (*p* < 0.05). No multiplicative or additive interaction was observed.

**Conclusion:**

Frailty and depression synergistically elevate digestive disease risk in aging populations, with bidirectional mediation underscoring their interdependence. Integrated interventions targeting mental health and geriatric vulnerability may mitigate disease burden.

## 1 Introduction

Digestive diseases pose a significant and growing global health challenge, contributing substantially to morbidity, healthcare costs, and disability worldwide (1). In the United States, these conditions affect over 40 million individuals, leading to millions of clinical visits annually and accounting for $119.6 billion in healthcare costs in 2018 (2). In China, up to 34.4% of adults reported chronic digestive disorders, underscoring their pervasive impact on aging populations (3). Beyond acute morbidity, digestive diseases such as chronic gastritis, inflammatory bowel disease, and nonalcoholic fatty liver disease are closely linked to long-term complications, including gastrointestinal cancers and metabolic dysfunction (4). The economic and societal burden of these conditions highlights the urgency of identifying modifiable risk factors and implementing preventive strategies to improve quality of life and reduce healthcare expenditures.

Depression, a prevalent mental health disorder, is increasingly recognized as a contributor to digestive pathology through bidirectional psychophysiological mechanisms (5). Chronic depression may exacerbate gastrointestinal inflammation, disrupt gut microbiota balance, and impair mucosal barrier function, potentially triggering or worsening conditions like irritable bowel syndrome and peptic ulcer disease (6, 7). Neuroendocrine dysregulation, including hypothalamic–pituitary–adrenal axis hyperactivity and elevated proinflammatory cytokines, has been implicated in this interplay (8). Ruan et al. (9) used Mendelian randomization analysis to suggest a potential causal relationship between depression and various gastrointestinal diseases, including irritable bowel syndrome, non-alcoholic fatty liver disease, alcoholic liver disease, gastroesophageal reflux disease, and chronic pancreatitis.

Frailty, a syndrome characterized by diminished physiological reserve and multisystem dysfunction, further compounds the vulnerability of older adults to digestive disorders (10, 11). Frail individuals often exhibit impaired nutrient absorption, reduced gastrointestinal motility, and compromised immune responses, which may predispose them to conditions such as dysphagia, gastroparesis, and *C. difficile* infections (12–14). Emerging evidence suggests that frailty and digestive diseases share common pathways, including chronic inflammation, oxidative stress, and mitochondrial dysfunction (15). However, the temporal relationship between frailty and digestive health remains underexplored, with few longitudinal studies addressing this interplay in aging Asian populations.

This study employs data from the China Health and Retirement Longitudinal Study (CHARLS) to examine the associations between depression, frailty, and their combined impact on the incidence of digestive diseases in older adults. By elucidating these relationships, this research aims to inform integrated care models that address both mental health and geriatric vulnerability, ultimately mitigating the dual burden of digestive and systemic comorbidities in aging societies.

## 2 Materials and methods

### 2.1 Study design and participants

This study is a secondary analysis utilizing data from the CHARLS, a nationally representative cohort of Chinese adults aged 45 years and older.^1^ The sample was drawn from 150 counties or districts and 450 villages across 28 provinces in China, covering the period from 2011 to 2020 (16).

**Figure 1:**
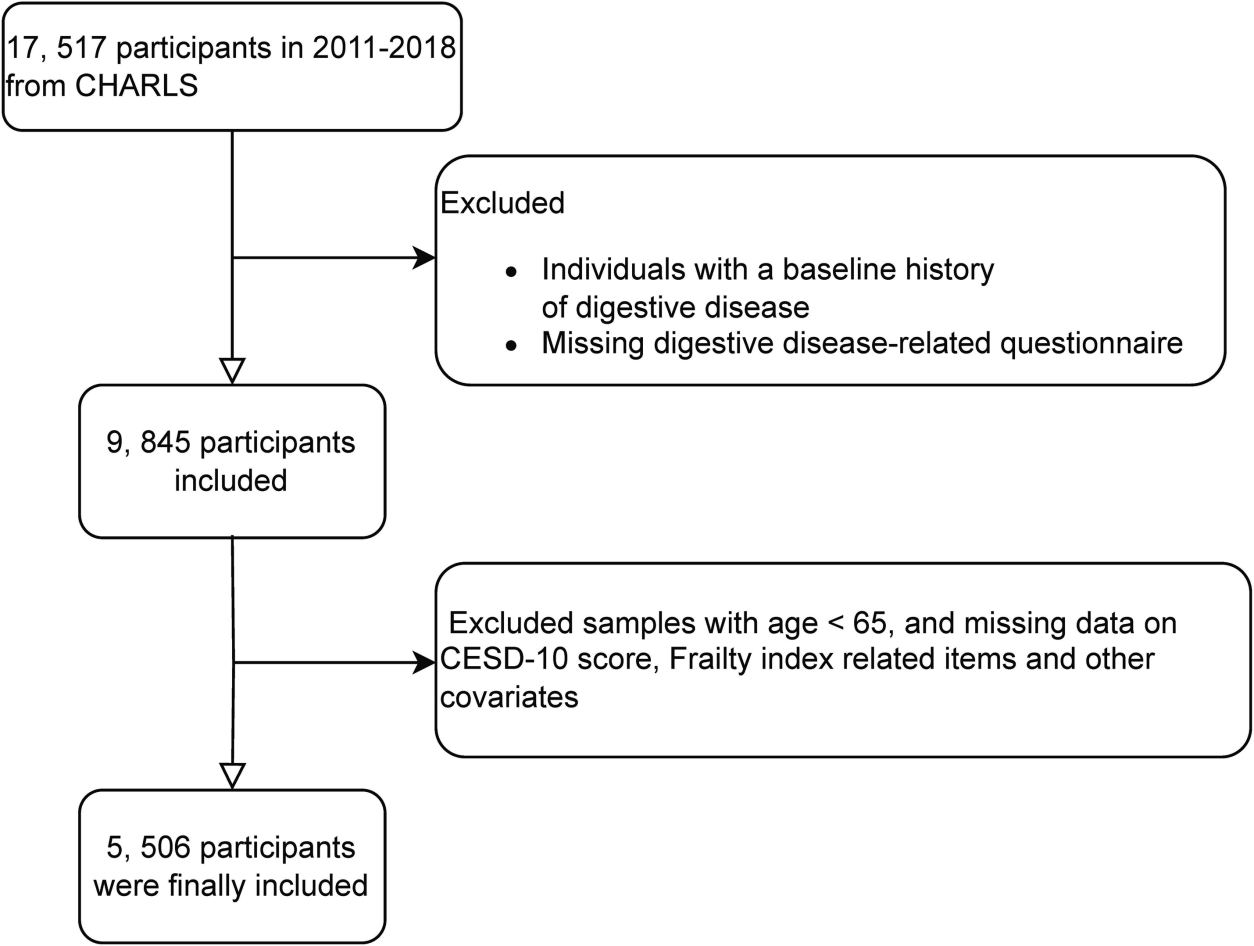
Flowchart of participant screening.

For this analysis, we utilized data from waves 1 to 4 of CHARLS (2011–2018), excluding wave 5 (2020) due to potential biases from the COVID-19 pandemic. Wave 1 in 2011 included 17,517 participants, from which individuals with baseline digestive disease or missing baseline digestive disease status were excluded. During follow-up (2013–2018), participants younger than 65 years or those with missing data on digestive disease status, frailty index items, CESD-10 scores, or other key covariates were further excluded. Additional exclusion criteria included missing data on educational attainment, alcohol consumption, hemoglobin levels, smoking status, diabetes mellitus, residential status, uric acid levels, heart disease status, and other essential covariates. These exclusions ensured dataset integrity, enhancing the accuracy and reliability of the statistical analysis (Figure 1).

### 2.2 Assessment of depression and frailty

Depressive symptoms were evaluated using the short version of the Center for Epidemiologic Studies Depression Scale (CES-D) in 2011 wave, a commonly employed self-reported tool for assessing depression in general populations (17). This scale comprises 10 items, each scored on a 4-point scale from 0 (rarely or not at all) to 3 (almost all the time). Participants with a total score of 10 or above were considered to have depressive symptoms (18, 19). The frailty index (FI) represents the cumulative burden of age-related health deficits (20, 21), encompassing 35 variables related to activities of daily living and instrumental activities of daily living, which include 11 tasks such as personal hygiene, dressing, and money management. It also includes physical function limitations (9 items), chronic diseases (9 items), psychological health indicators (5 items), and subjective assessments such as self-rated health (22), based on previous research using CHARLS to construct FI. Variables 1–35 were recoded to 0 (no deficit) and 1 (totally deficit) according to the corresponding criteria. When the number of missing items of a participant was > 20% (i.e., >7), his/her FI value was considered to be missing. While when the number of missing items was ≤20% (i.e., ≤7), the FI was equal to the sum of current health deficits divided by the number of non-missing items. Thus, FI was a continuous variable from 0 to 1, with higher FI indicating higher level of frailty in the participants. According to the previous consensus, participants were classified into a non-frail group (FI < 0.25) and frail group (FI ≥ 0.25) (23). Although a formal validation of the FI within the CHARLS cohort has not been performed, our construction approach is consistent with the published methodology. Indexes containing 30–40 variables are effective in predicting unfavorable health outcomes, according to previous research (24, 25). To ensure the suitability of the FI within the CHARLS dataset, we carefully selected 35 variables consistent with the standard deficit accumulation framework, covering domains recommended in prior literature (e.g., physical function, chronic disease burden, psychological symptoms, and subjective health). These variables were chosen based on data completeness, conceptual relevance, and prior usage in CHARLS-based studies. Furthermore, we excluded participants with more than 20% missing values in FI items, following accepted thresholds to maintain internal consistency and reduce measurement bias. This approach ensures that the derived FI reflects cumulative health deficits in a reliable and reproducible manner within the CHARLS population.

### 2.3 Assessment of new-onset digestive disease and their follow-up time

Digestive disease status was evaluated through participant interviews, including questions such as: “Have you been diagnosed with stomach or other digestive diseases (except for tumor or cancer) by a doctor?” The onset of digestive disease was recorded as the time of the initial diagnosis.

The incidence of digestive disease was assessed under different scenarios. For participants who did not report digestive disease at their most recent follow-up, the event time was calculated as the interval between the last survey year and the baseline year. For those who developed digestive disease, the timing was determined based on the difference between the earliest reported onset year and the baseline year (26).

### 2.4 Covariate

Based on prior research and expert recommendations, potential confounders and effect modifiers at baseline were identified, including age, sex (male or female), waist circumference, residence (urban or rural), and education level (less than high school, high school, or college). Clinical markers such as uric acid, creatinine, hemoglobin, blood lipids, and glucose were measured in the laboratory. Additionally, heart disease, dyslipidemia, hypertension, and diabetes mellitus were assessed using a standardized questionnaire that asked whether participants had ever been diagnosed with these conditions by a physician (27). Alcohol drinking status was classified into two distinct categories as ever/present or never. Smoke status was defined as former smoke but now quit, still smoke and never smoke (28, 29).

### 2.5 Statistical analysis

Data were presented as mean ± standard deviation (SD) for continuous variables with a normal distribution and as median with interquartile range for those with a non-normal distribution. Categorical variables were reported as counts and percentages. Group comparisons of baseline characteristics were conducted using the chi-squared test for categorical variables, analysis of variance (ANOVA) for normally distributed continuous variables, and the Kruskal–Wallis rank-sum test for non-normally distributed variables (30).

**Figure 2:**
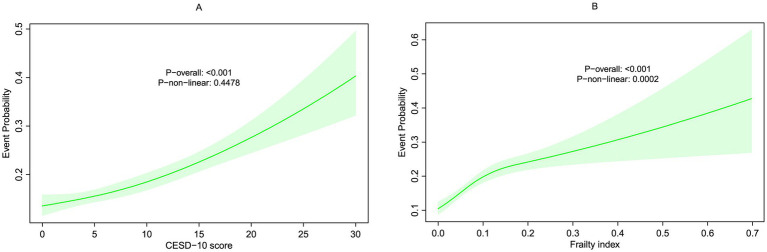
Restricted cubic spline (RCS) for the association between **(A)** CESD-10 score and **(B)** frailty index with the risks of new-onset digestive disease.

We calculated the follow-up person-time for each participant, starting from the baseline survey (2011–2012) until the occurrence of a digestive disease diagnosis or the end of the follow-up period (2017–2018), whichever came first. Cox proportional hazards regression models were employed to estimate hazard ratios (HRs) and 95% confidence intervals (CIs) for outcomes associated with depression and frailty. Three models were constructed: Model 0 (unadjusted); Model 1, adjusted for sex, waist circumference, smoking, and alcohol consumption; and Model 2, which included the adjustments from Model 1, plus uric acid, creatinine, hemoglobin, residence, heart disease, and HDL cholesterol. Additionally, 3-knot restricted cubic spline (RCS) regression was applied to examine potential nonlinear relationships in Figures 2A,B.

**Figure 3:**
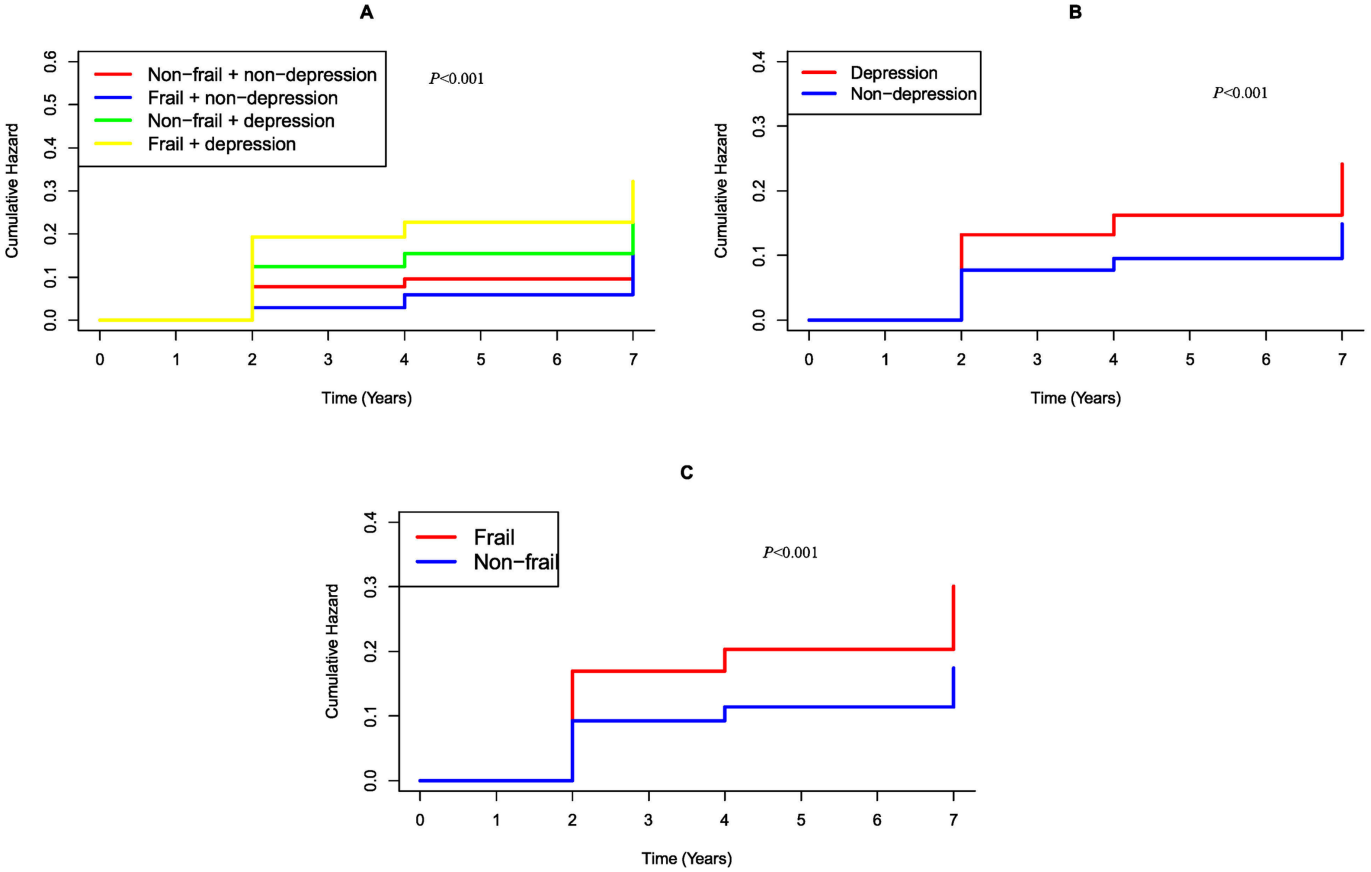
Kaplan Meier plot of digestive disease by CESD-10 score and frailty index subgroups. **(A)** Categorized by joint variable of CESD-10 score and frailty index; **(B)** Categorized by CESD-10 score; **(C)** Categorized by frailty index.

To assess the combined effects of frailty and depression on digestive disease, participants were categorized into four groups based on frailty status (frail vs. non-frail) and depressive status (depression vs. non-depression). Hazard ratios (HRs) for digestive disease incidence were calculated using the non-frail, non-depression group as the reference. Kaplan–Meier survival curves were generated to estimate median digestive disease-free survival (Figures 3A–C), and multivariable Cox regression was performed to identify risk factors (Table 1).

**Table 1:** Risk classification of new-onset digestive disease based on frailty index and depression by multiple Cox regression analysis.

Variables	Model 0	Model 1^a^	Model 2^b^
Frailty index	24.50 (12.98,46.24)^***^	20.91 (10.84,40.33)^***^	17.21 (8.65,34.24)^***^
Non-frail	ref	ref	ref
Frail	1.87 (1.47,2.39)^***^	1.78 (1.40,2.27)^***^	1.66 (1.29,2.12)^***^
CESD-10 score	1.05 (1.04,1.06)^***^	1.04 (1.03,1.05)^***^	1.04 (1.03,1.05)^***^
Non-depression	ref	ref	ref
Depression	1.72 (1.52,1.95)^***^	1.64 (1.45,1.87)^***^	1.62 (1.42,1.84)^***^
Joint variable			
Q1	ref	ref	ref
Q2	1.17 (0.52,2.61)	1.11 (0.49,2.48)	0.99 (0.44,2.21)
Q3	1.64 (1.44,1.87)^***^	1.57 (1.37,1.79)^***^	1.55 (1.36,1.77)^***^
Q4	2.44 (1.89,3.16)^***^	2.31 (1.78,2.99)^***^	2.16 (1.66,2.81)^***^

^a^Model 1 adjusted for sex, waist, smoke, alcohol drink. ^b^Model 2 adjusted for sex, waist, smoke, alcohol drink, uric acid, creatinine, hemoglobin, residence, heart disease and HDL. ^***^p < 0.001. Q1, Non-frail + non-depression; Q2, Frail + non-depression; Q3, Non-frail + depression; Q4, Frail + depression.

Mediation and interaction analyses were conducted to explore the direct and indirect effects of depression on digestive disease via an elevated FI. Additionally, the mediating role of depression in the frailty-digestive disease relationship was assessed. All statistical analyses were performed in R (version 4.2.1). Mediation analysis was conducted using the “mediation” and “charlsR” packages by bootstrap, while Cox regression utilized the “survival” package. A two-sided *p*-value of <0.05 was considered statistically significant (31).

## 3 Results

### 3.1 Study participants and baseline characteristics

The final cohort comprised 5,506 adults, including 988 participants diagnosed with new-onset digestive diseases (Table 2). Compared to the non-digestive disease group, individuals with digestive diseases exhibited a higher prevalence of FI and elevated CESD-10 scores. Additionally, they showed lower baseline BMI and hemoglobin levels, alongside a greater proportion of comorbid heart disease. No significant differences were observed in age, educational attainment, residential distribution, or fasting glucose levels between the two groups (all *p* > 0.05).

**Table 2:** Baseline characteristics of participants.

Variables	Overall (*n* = 5, 506)	No digestive diseases (*n* = 4, 518)	Digestive diseases (*n* = 988)	*p* value
Age (years)	58.35 ± 9.07	58.27 ± 9.03	58.72 ± 9.24	0.17
Sex (Male %)	2,544 (46.20)	2,144 (47.45)	400 (40.49)	<0.0001
BMI (kg/m^2^)	23.80 ± 3.95	23.86 ± 3.87	23.50 ± 4.26	0.01
Hemoglobin (g/dL)	14.42 ± 2.24	14.48 ± 2.25	14.16 ± 2.19	<0.0001
Education (%)				0.66
Less Than High School	4,942 (89.76)	4,051 (89.66)	891 (90.18)	
College	68 (1.24)	54 (1.20)	14 (1.42)	
High School	496 (9.01)	413 (9.14)	83 (8.40)	
Residence				0.34
Rural	3,615 (65.66)	2,953 (65.36)	662 (67.00)	
Urban	1,891 (34.34)	1,565 (34.64)	326 (33.00)	
Glucose (mg/dL)	110.33 ± 35.88	110.64 ± 36.42	108.91 ± 33.30	0.15
Creatinine (mg/dL)	0.77 ± 0.19	0.78 ± 0.19	0.76 ± 0.19	<0.01
Uric acid (mg/dL)	4.44 ± 1.25	4.47 ± 1.26	4.33 ± 1.22	<0.001
Waist (cm)	84.89 ± 12.49	85.10 ± 12.49	83.94 ± 12.43	<0.01
Dyslipidemia (yes%)	2,251 (40.88)	1,843 (40.79)	408 (41.30)	0.80
TC (mg/dL)	193.20 ± 38.18	193.08 ± 38.24	193.74 ± 37.90	0.62
HDL-C (mg/dL)	50.72 ± 15.13	50.51 ± 15.03	51.67 ± 15.56	0.03
LDL-C (mg/dL)	116.12 ± 35.06	116.12 ± 34.97	116.11 ± 35.49	0.99
TG (mg/dL)	133.48 ± 96.40	133.96 ± 96.98	131.25 ± 93.69	0.41
Smoke status (%)				<0.01
Former, now quit	450 (8.17)	354 (7.84)	96 (9.72)	
Never	3,383 (61.44)	2,754 (60.96)	629 (63.66)	
Current	1,673 (30.39)	1,410 (31.21)	263 (26.62)	
Alcohol drink (%)				<0.01
No	3,645 (66.20)	2,954 (65.38)	691 (69.94)	
Yes	1,861 (33.80)	1,564 (34.62)	297 (30.06)	
Frailty index	0.09 ± 0.08	0.09 ± 0.08	0.12 ± 0.09	<0.0001
Frail	236 (4.29)	165 (3.65)	71 (7.19)	
Non-frail	5,270 (95.71)	4,353 (96.35)	917 (92.81)	
Diabetes Mellitus (%)	804 (14.60)	659 (14.59)	145 (14.68)	0.98
Heart disease (%)	493 (8.95)	366 (8.10)	127 (12.85)	<0.0001
Hypertension (%)	2,225 (40.41)	1,839 (40.70)	386 (39.07)	0.36
CESD-10 score	7.76 ± 6.05	7.39 ± 5.83	9.42 ± 6.70	<0.0001
Depression (%)	3,670 (66.65)	3,125 (69.17)	545 (55.16)	<0.0001
Follow up time (years)	6.46 ± 1.51	7.00 ± 0.00	3.97 ± 2.29	<0.0001

BMI, body mass index; TC, total cholesterol; HDL, high-density lipoprotein; LDL, low-density lipoprotein; TG, triglycerides; CESD-10, the 10-item Center for Epidemiologic Studies Depression Scale.

### 3.2 Correlation between depression, frailty, and new-onset digestive disease

RCS analyses (Figures 2A,B) demonstrated a linear positive association between CESD-10 scores and digestive disease risk (*P* overall < 0.001). The FI displayed a non-linear relationship with risk (*P* overall < 0.001, *P* non-linear = 0.0002), where risk escalated sharply when FI > 0.1.

### 3.3 Associations of depression, frailty, and their combined effect on digestive disease

Multivariable cox regression (Table 1) revealed that both frailty (HR = 1.66, *p* < 0.001) and depression (HR = 1.62, *p* < 0.001) independently predicted digestive disease risk after adjusting for multiple confounders. A joint analysis highlighted a graded increase in risk: participants with both frail and depression (Q4) faced the highest hazard (adjusted HR = 2.16, *p* < 0.001), compared to non-frail and non-depression group (Q1), surpassing risks observed in isolated frailty or depression subgroups.

### 3.4 Mediation analyses of frailty and depression in digestive disease

**Figure 4:**
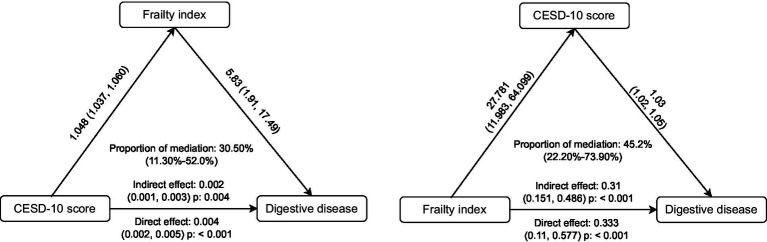
Mediation analyses of CESD-10 score and frailty index on new-onset digestive disease.

Mediation analysis (Figure 4) indicated bidirectional effects. Frailty mediated 30.50% of the association between depression and digestive diseases (indirect effect *p* = 0.004), while depression mediated 45.20% of the frailty-digestive disease link (indirect effect *p* < 0.001), underscoring their interconnected roles in disease pathogenesis.

### 3.5 Interactive effects of frailty and depression on digestive disease risk

No significant multiplicative (HR = 1.41, 95% CI: 0.60–3.29) or additive interactions (RERI = 0.63, 95% CI: −0.34–1.6) were detected between frailty and depression (Table 3). These results suggest that their combined risk operates additively rather than synergistically.

**Table 3:** Interaction analysis of depression and frailty on new-onset digestive disease.

Variables	HR [95% CI]
Multiplicative scale	1.41 [0.60, 3.29]
RERI	0.63 [−0.34, 1.6]
AP	0.29 [−0.13, 0.71]
SI	2.17 [0.44, 10.73]

## 4 Discussion

Our study suggests that both frailty and depression independently contribute to the risk of digestive disease, with potentially compounding effects when these factors co-occur. Previous research has demonstrated a strong association between depression and gastrointestinal disorders, including irritable bowel syndrome (IBS), gastroesophageal reflux disease, and inflammatory bowel disease (32–34). Lee et al. (35) demonstrated significant associations of depression with functional dyspepsia, IBS, reflux esophagitis, peptic ulcer disease, and colorectal/gastric adenoma or carcinoma. Furthermore, Yun et al. (36) identified constipation as a potential independent risk factor or prodromal manifestation of depressive disorders. Mechanistically, the gut-brain axis plays a crucial role in this relationship, with depression-induced dysregulation of the hypothalamic–pituitary–adrenal axis leading to neuroendocrine hormones change and altered gut microbiota composition (37, 38). The gut microbiota was thought to significantly influence the metabolism of tryptophan, which is linked to the development of clinical depression (39). Studies on germ-free mice have shown that they exhibit higher serum tryptophan levels and lower blood serotonin concentrations compared to conventionally colonized mice. This suggests that the expression of tryptophan hydroxylase in the intestines may be diminished in germ-free mice (40, 41). Compared to healthy controls, individuals with depression exhibit altered intestinal microbial composition, characterized by reduced diversity and depletion of anti-inflammatory taxa (e.g., Lactobacillus, Bifidobacterium) (42). Preclinical studies demonstrate that fecal microbiota transplantation from depression donors induces depression-like behaviors and gastrointestinal dysfunction (e.g., visceral hypersensitivity, impaired motility) in recipient rodents, while probiotic interventions reverse these phenotypes (43).

Frailty, a geriatric syndrome characterized by decreased physiological reserves and increased vulnerability to stressors, has also been implicated in digestive disease risk. Prior observational studies have found that frail older adults are more likely to experience chronic constipation, delayed gastric emptying, and malabsorption syndromes (44–46), with some randomized controlled trials suggesting that frailty-targeted interventions, such as nutritional supplementation and resistance training, may improve digestive health outcomes (47, 48). The underlying mechanisms linking frailty and digestive diseases likely involve chronic low-grade inflammation, oxidative stress, and metabolic dysfunctions, including insulin resistance and dyslipidemia (15, 49). Additionally, aging-related declines in anabolic hormones, such as insulin-like growth factor-1, may contribute to muscle wasting and impaired gastrointestinal motility (50). Our findings align with this body of evidence, showing that frailty was independently associated with digestive disease risk, with a non-linear relationship indicating a sharp increase in hazard ratios when the frailty index exceeded a certain threshold.

Although evidence on the synergistic interaction between frailty and depression in digestive disease progression remains limited, emerging data indicate their compounded risk in mortality. In the Kashiwa Cohort Study, Hamada et al. demonstrated that frail older adults with concurrent depressive symptoms exhibited a 4.34-fold higher mortality risk compared to non-frail counterparts without depression, underscoring the critical interplay of psychosocial and physiological vulnerabilities in adverse outcomes (51). Additionally, our study identified bidirectional mediation between frailty and depression in the pathogenesis of digestive diseases. Mechanistically, as shown in the Rotterdam Study, where depression-driven HPA axis hyperactivity and mitochondrial dysfunction exacerbated frailty, impairing gut motility and possibly increasing risks of gastroparesis and ischemic colitis (52). Conversely, frailty-associated inflammation (e.g., IL-6, TNF-*α*) disrupting gut microbiota and elevating intestinal permeability, which amplifies depressive symptoms and visceral hypersensitivity in IBS (53, 54). Additionally, a systematic review has identified a bidirectional relationship between frailty and depression (55), potentially due to shared pathophysiological mechanisms, such as chronic inflammation. Inflammatory biomarkers, including IL-6 and C-reactive protein, may act as intermediaries in this connection (56).

Our findings carry substantial clinical implications. Given the broad spectrum of digestive diseases, early screening and preventive interventions are crucial for frail and depressed individuals. Routine gastrointestinal evaluations, including upper endoscopy, colonoscopy, and abdominal imaging studies, should be considered in high-risk populations. Additionally, monitoring dietary habits, bowel movement patterns, and nutritional status may facilitate early detection and timely management of digestive disorders. Preventive strategies should not only focus on digestive health but also address underlying frailty and depression through multidisciplinary interventions, including pharmacologic therapy, rehabilitation programs, and psychological counseling. Implementing targeted interventions in these vulnerable populations may help reduce the burden of digestive diseases and improve overall health outcomes in aging adults.

Although no significant interaction between frailty and depression was observed, the presence of bidirectional mediation underscores the complex interrelationship between these factors. This apparent discrepancy is statistically plausible, as interaction and mediation analyses serve distinct purposes. Interaction analysis assesses whether the effect of one exposure on the outcome is modified by the presence of another, indicating effect modification, whereas mediation analysis explores whether an exposure influences the outcome indirectly through a mediator. The absence of a significant interaction does not preclude the existence of meaningful mediation pathways. In our study, both approaches were employed to provide a comprehensive understanding of how frailty and depression contribute to digestive disease risk. Interaction analysis enabled us to examine potential synergistic or antagonistic effects, while mediation analysis elucidated the indirect causal mechanisms linking the two factors. The integration of these complementary methods enhances the interpretability of our findings and reflects the multidimensional nature of psychosocial and physiological vulnerability in aging populations.

This study has several strengths. First, the use of CHARLS data allows for a large, nationally representative sample with longitudinal follow-up, providing robust evidence on frailty, depression, and digestive disease risk. Second, the mediation analysis offers novel insights into the bidirectional relationship between frailty and depression in digestive disease development. However, several limitations should be noted. First, digestive disease diagnoses were based on self-reported data, which may introduce recall bias. Second, while extensive confounders were considered, unmeasured factors such as dietary habits, medication use, or microbiome composition could still influence the observed associations. Finally, the observational nature of the study precludes establishing causality. Future studies with objective clinical assessments and randomized controlled trials are warranted to validate these findings.

Further research is needed to elucidate the biological mechanisms linking frailty, depression, and digestive disease risk. Investigations into inflammatory pathways, gut microbiota alterations, and neuroendocrine dysregulation may provide mechanistic insights. Additionally, neuroimaging studies could explore potential structural changes in the central nervous system contributing to frailty and depression-related gastrointestinal dysfunction. Clinical trials assessing the effectiveness of multidisciplinary interventions—combining nutritional support, physical activity, psychological therapy, and gut-targeted treatments—may offer evidence-based strategies to reduce digestive disease burden in older adults. Addressing these modifiable risk factors could pave the way for precision medicine approaches in geriatric gastroenterology.

## 5 Conclusion

This study demonstrates that frailty and depression independently and jointly elevate new-onset digestive disease risk in middle-aged and older Chinese adults, with the highest hazard observed in comorbid cases. Mediation analyses revealed bidirectional pathways: frailty mediated 30.5% of depression’s effect, while depression mediated 45.2% of frailty’s impact. No synergistic interaction was detected, suggesting additive effects. These findings highlight the need for comprehensive, multidisciplinary interventions—such as psychological counseling, nutritional support, physical exercise programs, and routine gastrointestinal screening—targeting both mental health and geriatric frailty. Such integrative strategies may help mitigate the burden of digestive diseases and improve overall health outcomes in aging populations. Future research should elucidate biological mechanisms and validate holistic care models in aging populations.

## Data Availability

The datasets presented in this study can be found in online repositories. The names of the repository/repositories and accession number(s) can be found at: http://charls.pku.edu.cn/en.

## Footnotes

^1^ http://charls.pku.edu.cn/
